# An Atypical Case of Concurrent Emphysematous Cystitis and Osteomyelitis in a Patient With Metastatic Cancer

**DOI:** 10.7759/cureus.93508

**Published:** 2025-09-29

**Authors:** Kanwal Jussa, Widad Labban, Mary Tran, John Greene

**Affiliations:** 1 Internal Medicine, Nova Southeastern University Dr. Kiran C. Patel College of Osteopathic Medicine, Tampa, USA; 2 Internal Medicine, Moffitt Cancer Center, Tampa, USA

**Keywords:** emphysema cystitis, emphysematous hemorrhagic cystitis (ehc), emphysematous infection, emphysematous osteomyelitis, infectous diseases, metasatic metaplastic breast cancer, osteo-myelitis

## Abstract

Emphysematous cystitis (EC) is a rare, gas-forming bladder wall infection, most commonly caused by *Escherichia coli* *(E. coli)* and typically seen in diabetic or immunocompromised patients. We report a case of a 50-year-old female with breast cancer undergoing neoadjuvant chemotherapy who presented with hematuria, nausea, and vomiting. Imaging and laboratory workup revealed EC and concurrent osteomyelitis caused by *Pseudomonas aeruginosa (P. aeruginosa)*. She was treated with extended antibiotic therapy, leading to stabilization of the osteomyelitis. This report highlights the unusual role of *P. aeruginosa*, a less common gas-producing organism, in the pathogenesis of EC. We also review two additional cases of EC in oncology patients undergoing chemotherapy, with the underlying microbe being *Klebsiella pneumoniae (K. pneumoniae), *and treated with appropriate antibiotics. These cases further emphasize the significance of EC in patients with metastatic cancer undergoing chemotherapy to prompt early investigation and guide treatment.

## Introduction

Emphysematous cystitis (EC) is an uncommon but potentially life-threatening urinary tract infection (UTI) characterized by gas formation within the bladder wall and lumen by gas-forming organisms such as *Escherichia coli (E. coli), Klebsiella pneumoniae (K. pneumoniae)*, and *Streptococcus species*. EC occurs most frequently in diabetic patients and is reported twice as often in women as in men [1,2]. While a history of diabetes mellitus is the most common risk factor, additional associations include a history of neurogenic bladder, recurrent UTIs, prior pelvic surgeries, chemotherapy treatments, and malnutrition [2,3]. The pathogenesis is complex and poorly understood [4]; however, it often involves impaired host immunity, with gas production possibly resulting from bacterial fermentation of glucose or, less commonly, proteins [5,6]. The clinical presentation of EC varies widely. While some patients may be asymptomatic or present with milder symptoms resembling uncomplicated cystitis, others may develop more severe manifestations, including pneumaturia, peritonitis, or septic shock. Macroscopic hematuria is a commonly noted symptom, as well as complaints of abdominal pain, and symptoms of classic UTIs (i.e., dysuria, increased frequency, urgency) [1]. 

CT of the abdomen and pelvis is the preferred imaging modality for diagnosis, offering high sensitivity and specificity for detecting intramural or intraluminal gas, and distinguishing EC from similar conditions such as bladder fistulas or emphysematous pyelonephritis [1,3]. Although many cases of EC follow a relatively benign course, severe complications do occur, such as bladder rupture, abscess formation, and septic shock. EC carries a mortality of 10.4%, necessitating timely diagnosis and prompt initiation of antibiotic therapy [3]. Of particular concern, these challenges are amplified in immunocompromised and oncology patients, where overlapping symptoms, treatment-related cytopenias, and increased susceptibility to infection may complicate both recognition and management.

While EC itself is rare, its co-occurrence with osteomyelitis of the pubic symphysis (OP) is exceedingly uncommon, with only a few cases reported in the literature. OP, described as infection and subsequent inflammation of the symphysis pubis, accounts for only 2% of hematogenous osteomyelitis (OM) [7] and less than 1% of all osteomyelitis cases [8]. Presentation is often nonspecific, ranging from pelvic pain to fever, making early diagnosis critical to avoid severe complications and surgical intervention [7,8]. Importantly, concurrent EC and OP in oncology patients has rarely been described. This report highlights such a scenario, adding to the limited literature and encouraging awareness of such presentation in immunocompromised patients. We present a case of a 50-year-old female undergoing carboplatin and paclitaxel chemotherapy for triple-negative breast cancer who developed EC with concurrent osteomyelitis of the pubic symphysis and bilateral pubic rami.

## Case presentation

The patient was a 50-year-old female with a complex medical history, including triple-negative invasive ductal carcinoma of the left breast (Grade 3), who was receiving weekly neoadjuvant carboplatin and paclitaxel (Carbo-Taxol) chemotherapy. Her history included Hodgkin’s lymphoma treated with chemotherapy and mantle field radiation decades earlier, morbid obesity (BMI >50 kg/m²), pulmonary embolism on apixaban, osteoarthritis, remote tobacco use, and allergies to levofloxacin, penicillin, and sulfonamides. She presented to the cancer center with a six-day history of gross hematuria with large clots, intractable nausea and vomiting despite home antiemetics, diarrhea, periumbilical abdominal pain, and right hip pain following her fourth chemotherapy cycle. In addition, she reported a 40-pound unintentional weight loss over three weeks, attributed to side effects of the chemotherapy regimen. She had experienced similar urinary symptoms two months prior and was empirically treated with a seven-day course of nitrofurantoin for a UTI.

On presentation, her vital signs were within normal limits. Physical examination was notable for obesity but otherwise unremarkable, with no costovertebral angle tenderness or suprapubic tenderness appreciated at the time. Lab findings, including complete blood count with differential count, chemistry panel, iron study panel, folate level, vitamin B12 level, and inflammatory marker tests, are displayed in Tables 1-4, respectively. A blood culture was not obtained at this time.

**Table 1:** Hemogram with differential count (%), obtained on the day of admission, displaying patient’s lab values compared to the reference range ^†^Reference range values obtained from The American Board of Internal Medicine Reference Ranges, updated January 2025

Hemogram with differential count (%)		
Test	Patient’s lab value	Reference range^†^
Leukocyte count	4,910/μL	4000–11,000/μL
Erythrocyte count	2.76 mil/μL	4.2–5.9 million/μL
Hemoglobin	8.4 g/dL	Female: 12–16 g/dL; male: 14–18 g/dL
Hematocrit	26.9%	Female: 37%–47%; male: 42%–50%
Mean corpuscular volume	97.5 fL	80–98 fL
Mean corpuscular hemoglobin	30.4 pg	28–32 pg
Mean corpuscular hemoglobin concentration	31.2 g/dL	33–36 g/dL
Platelet count	289,000/μL	150,000–450,000/μL
Mean platelet volume	9.6 fL	7–9 fL
Neutro, auto	63%	50%–70%
Eos, auto	1.0%	0%–3%
Basophil, auto	1.2%	0%–1%
Mono, auto	5.1%	0%–6%
Lymph, auto	28.7%	30%–45%

**Table 2:** Routine chemistry panel (serum), obtained on the day of admission, displaying patient’s lab values compared to the reference range ^†^Reference range values obtained from The American Board of Internal Medicine Reference Ranges, updated January 2025

Routine chemistry, serum		
Test	Patient’s lab value	Reference range^†^
Sodium	137 mEq/L	136–145 mEq/L
Potassium	3.0 mEq/L	3.5–5.0 mEq/L
Chloride	98 mEq/L	98–106 mEq/L
Total CO_2_	33 mEq/L	23–30 mEq/L
Glucose level	85 mg/dL	70–99 mg/dL
Blood urea nitrogen (BUN)	6 mg/dL	8–20 mg/dL
Creatinine	0.4 mg/dL	Female: 0.50–1.10 mg/dL; male: 0.70–1.30 mg/dL
Est. GFR	>60 mL/min/1.73m^2^	—
Calcium	7.2 mg/dL	8.6–10.2 mg/dL
Calcium corrected	8.5 mg/dL	8.6–10.2 mg/dL
Phosphorus	3.3 mg/dL	3.0–4.5 mg/dL
Total protein	5.2 g/dL	5.5–9.0 g/dL
Albumin	2.4 g/dL	3.5–5.5 g/dL
Total bilirubin	0.4 mg/dL	0.3–1.0 mg/dL
Alkaline phosphatase	91 U/L	30–120 U/L
Aminotransferase, serum aspartate (AST, SGOT)	46 U/L	10–40 U/L
Aminotransferase, serum alanine (ALT, SGPT)	23 U/L	10–40 U/L
Magnesium level	1.9 mg/dL	1.6–2.6 mg/dL
Lactic acid	0.8 mmol/L	0.7–2.1 mmol/L
Procalcitonin	0.21 ng/mL	≤0.10 ng/mL

**Table 3:** Serum iron studies and vitamin levels, obtained on the day of admission, displaying patient’s lab values compared to the reference range ^†^Reference range values obtained from The American Board of Internal Medicine Reference Ranges, updated January 2025

Iron studies and vitamin levels panel, serum		
Test	Patient’s lab value	Reference range^†^
Folic acid level	<2.2 ng/mL	1.8–9.0 ng/mL
Iron	26 μg/dL	50–150 μg/dL
Iron-binding capacity, total	135 μg/dL	250–310 μg/dL
Transferrin saturation	19%	20%–50%
Ferritin	460 ng/mL	Female: 24–307 ng/mL; male: 24–336 ng/mL
Vitamin B12 Level	508 pg/mL	200–800 pg/mL

**Table 4:** CRP and ESR levels obtained on the day of admission, showing patient’s lab values compared to reference ranges † Reference range values obtained from The American Board of Internal Medicine Reference Ranges, updated January 2025.

Inflammatory markers, serum		
Test	Patient’s lab value	Reference range^†^
C-reactive protein, HS (CRP)	4.28 mg/dL	≤0.8 mg/dL
Erythrocyte sedimentation rate (ESR)	60 mm/hr	Female: 0–20 mm/hr; male: 0–15 mm/hr

Urinalysis was positive for leukocyte esterase. Stool PCR testing was positive for *Clostridioides difficile (C. difficile)* but antigen-negative. Thus, no treatment was given as the diarrhea was most likely secondary to the chemotherapy. 

**Figure 1:**
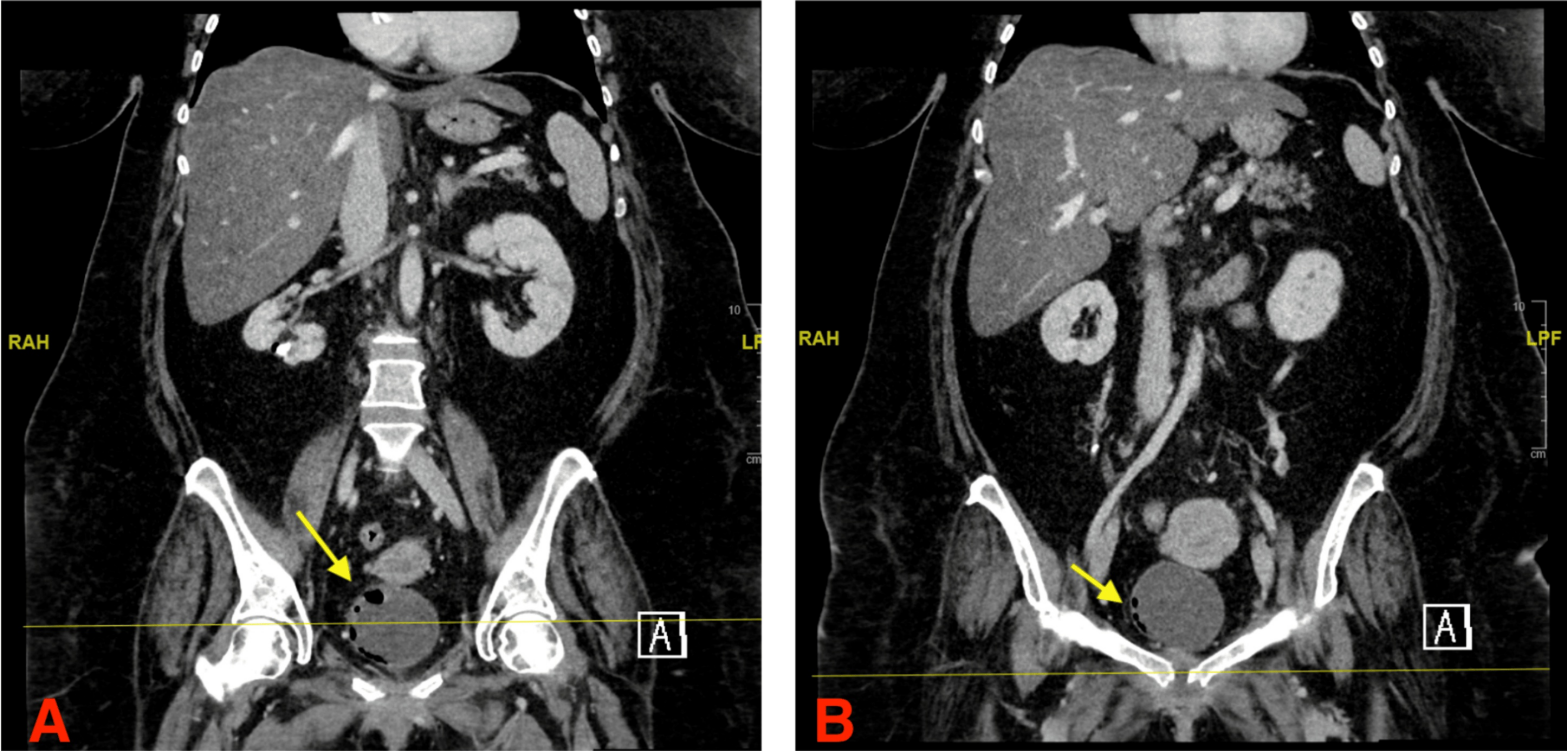
CT scan images (A and B) show pneumatosis of the urinary bladder wall (depicted by yellow arrows) The findings are compatible with emphysematous cystitis CT: computed tomography

A CT scan of the patient’s abdomen and pelvis was significant for irregularity of the pubic symphysis (concerning for osteomyelitis and septic arthritis), EC, and gas within the lower right renal calyx and bladder wall (Figure 1). Subsequent MRI of the abdomen and pelvis (with and without contrast) confirmed intense heterogeneous enhancement of the symphysis pubis and bilateral pubic rami (consistent with septic arthritis and osteomyelitis), EC, and marked diffuse hepatic steatosis.

Empiric intravenous (IV) ceftriaxone was initiated to target common uropathogens such as *E. coli* and *Klebsiella* species. Urine cultures collected after initiation of antibiotics showed only mixed flora, consistent with vaginal contaminants. Further testing for BK virus (human polyomavirus 1) and adenovirus was negative. BK virus and adenovirus testing were considered, as these viruses are possible infectious causes of hematuria [9]. During hospitalization, the patient underwent a CT-guided fine-needle aspiration biopsy of the pubic symphysis. The culture of the specimen grew *Pseudomonas aeruginosa (P. aeruginosa)*, which was susceptible to cefepime. Accordingly, the antibiotic regimen was escalated to IV cefepime, 2 grams every 12 hours, planned for six weeks.

After a 10-day inpatient stay, the patient was discharged with instructions to continue IV cefepime (2 grams every 12 hours) through home health services, with follow-up arranged at the General Infectious Disease Clinic upon completion of the six-week course. The patient’s assessment (regarding the initial presentation) as of discharge was as follows: EC without severe septic illness and septic arthritis/osteomyelitis of the symphysis pubis and bilateral pubic rami. 

A repeat MRI obtained one month after discharge demonstrated findings consistent with persistent osteomyelitis of the symphysis pubis and bilateral pubic rami, with improvement of the EC. At that time, the patient denied hematuria, passage of clots, constipation, or diarrhea but reported ongoing nausea with fewer episodes of emesis. As symptoms and imaging suggested incomplete response, cefepime was extended for six more weeks at 6 g per day, with stewardship review confirming appropriate dosing. Despite therapy, follow-up imaging showed minimal improvement and rising inflammatory markers. Antibiotics were discontinued, and a bone biopsy was considered; however, the patient was deemed unsuitable for surgery. Subsequent imaging demonstrated stable chronic osteomyelitis. Her course was later complicated by the metastatic progression of breast cancer to the brain and lungs, and she passed away thereafter.

In addition to this primary case, we identified two additional patient cases that received a diagnosis of EC.

Case 2

**Figure 2:**
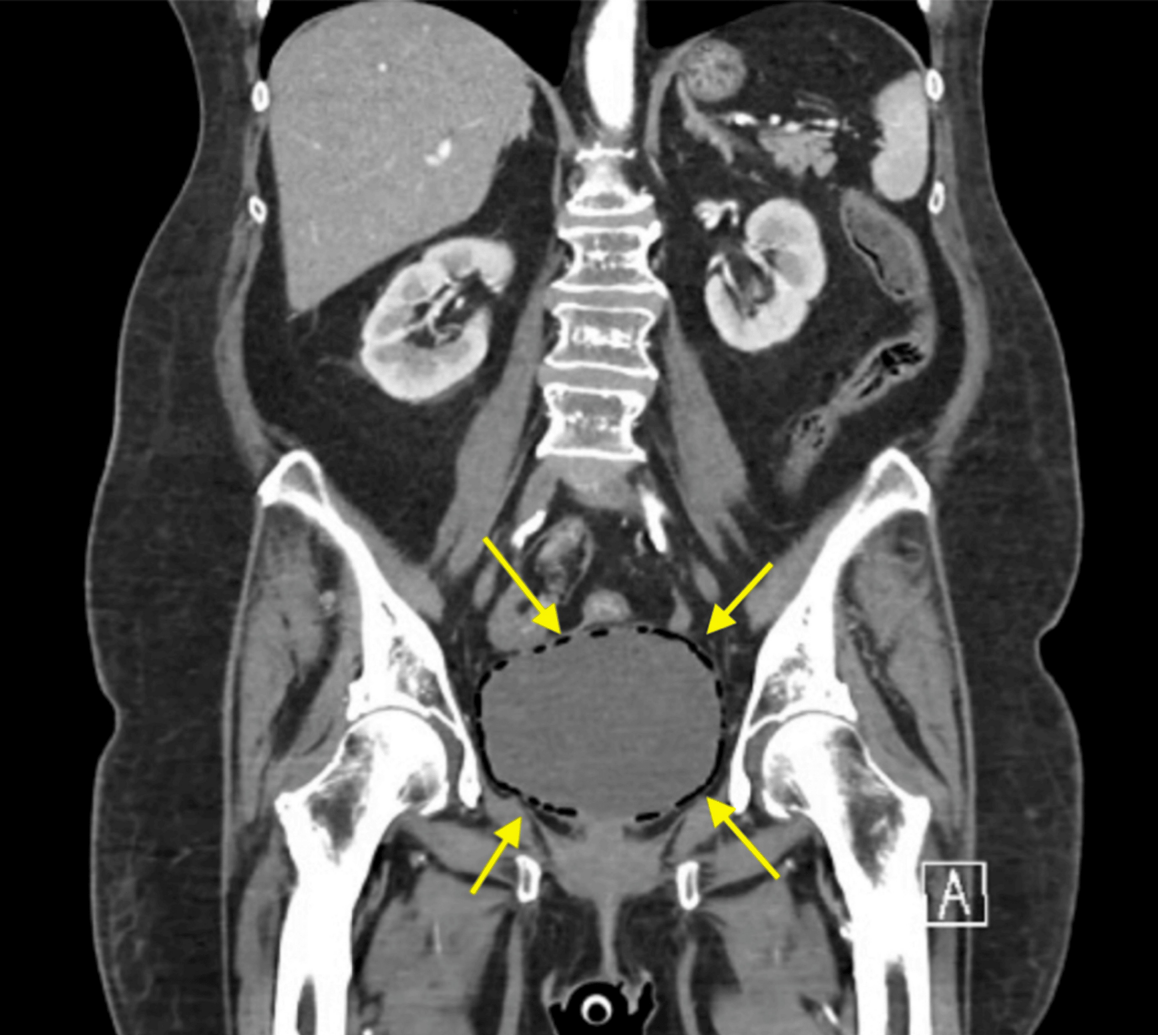
Extensive gas within the bladder wall in Case 2

This patient was a 67-year-old female with metastatic high-grade serous ovarian cancer on Carbo-Taxol with complaints of possible candida esophagitis, chronic dysuria, and new-onset urinary retention in the background of chronic prednisone use. During a prior admission, she had been treated for a UTI with *K. pneumoniae* that initially resolved with Augmentin. However, her symptoms shortly returned. A CT scan revealed EC, and urinalysis revealed glucosuria (Figure 2). Subsequently, she was treated with micafungin for esophagitis and ceftriaxone, a standard therapy for treating Gram-negative organisms. She was then discharged to a rehab facility where she continued the antibiotic treatment for a minimum of two weeks, with anticipated improvement with optimized glucose control. She is currently well and is following up with her gynecologist as she undergoes maintenance treatment of her cancer. This case represents a typical presentation of EC with the gas-forming *K. pneumoniae*.

Case 3

**Figure 3:**
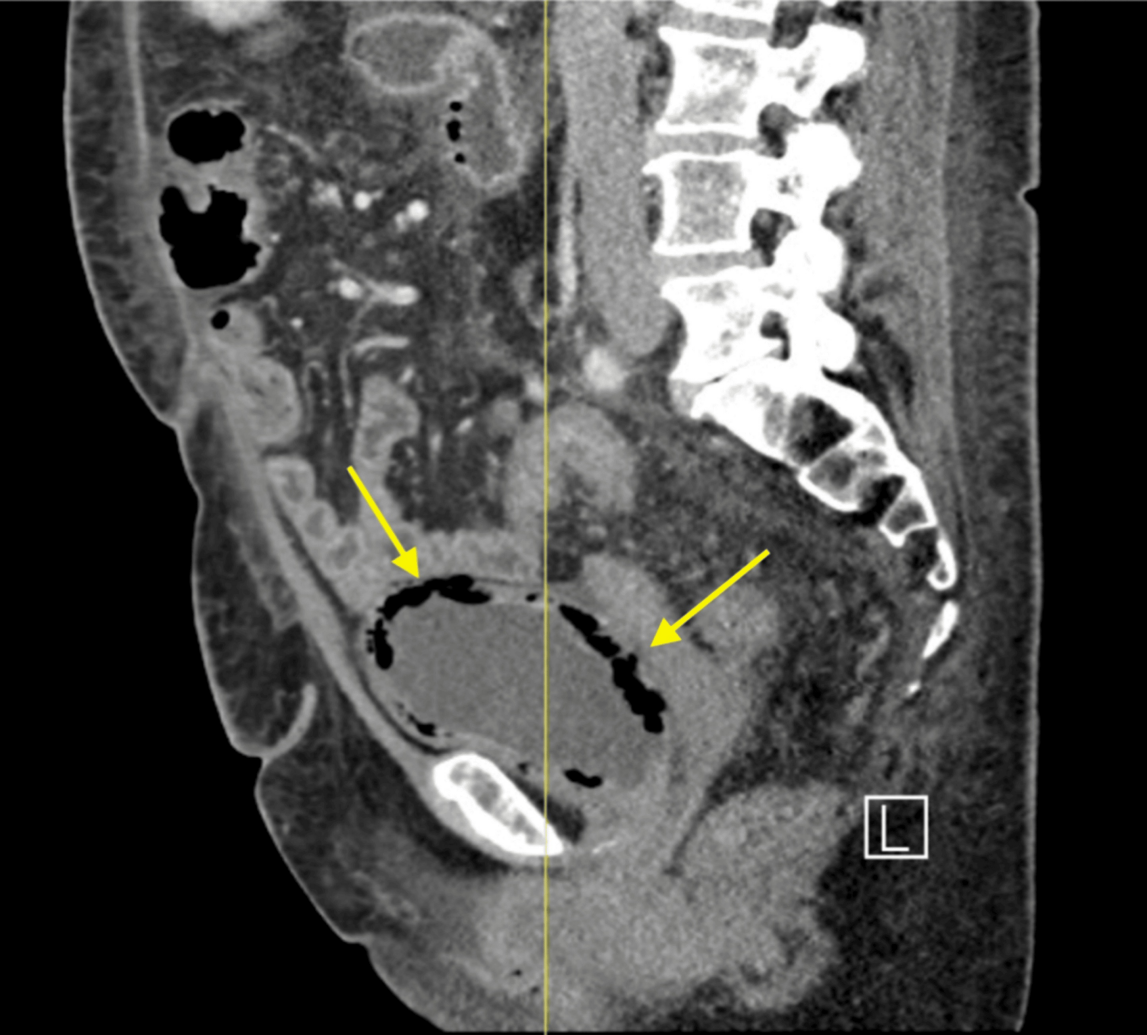
CT image displaying pneumatosis of the bladder wall consistent emphysematous cystitis in Case 3 CT: computed tomography

This case involves a 68-year-old female with a malignant islet cell tumor undergoing FOLFOX chemotherapy. Additional pertinent medical history includes a diagnosis of diabetes and chronic prednisone use, both risk factors for developing severe infection. Although she was asymptomatic, urine cultures grew *K. pneumoniae*, most likely due to her immunocompromised and diabetic status. Her CT imaging ultimately revealed extensive air within the bladder wall (Figure 3). The patient was started on empiric antibiotic treatment while sensitivities were pending. This patient later passed away due to progression of her cancer and ultimately sepsis due to a catheter infected with methicillin-susceptible *Staphylococcus aureus* (MSSA). This case suggests that EC may follow an indolent course, especially in immunocompromised or diabetic patients, and may be detected on imaging before the onset of symptoms.

## Discussion

Carbo-Taxol is commonly used in treating breast, ovarian, and lung cancers [10]. Carboplatin is often paired with paclitaxel due to their synergistic antitumor effects [11]. Carbo-Taxol treatment commonly results in myelosuppression along with peripheral neuropathy and gastrointestinal side effects. While hematuria appears infrequently as a side effect, carboplatin-induced hemorrhagic cystitis has been reported, likely due to urothelial irritation and sloughing [12].

Hemorrhagic cystitis remains an uncommon yet serious complication during carboplatin-based chemotherapy treatments. Diagnosis is made clinically by the presence of hematuria and confirmed with urinalysis and various imaging studies. Imaging findings of intramural gas in our patient suggested EC, which can arise in immunocompromised states, such as chemotherapy-induced neutropenia. While urine cultures are collected and results are pending, management of EC and HC begins with initiation of empirical antibiotic treatment, typically targeting Gram-negative bacteria such as *E. Coli *and *Klebsiella*. If urine cultures remain negative, management still includes continued broad-spectrum antibiotics, bladder irrigation, and close monitoring for any signs of improvement. In the case that a patient fails conservative medical management, surgery is the subsequent option, especially in the presence of severe complications, such as bladder rupture or extensive infection [13]. Surgical options include partial or total cystectomy as well as surgical debridement or cystostomy [14].

Septic arthritis and osteomyelitis of the pubic symphysis are extremely rare complications but have been increasingly documented in patients with a history of pelvic malignancy or prior surgery [15]. *Clostridium* is the primary cause of gas in tissue. However, *S. aureus, Escherichia coli, Klebsiella*, and *Enterococcus faecalis* are more commonly implicated in the genitourinary system, since anaerobic infections are rare in this region [16]. In a 2022 review of 13 patients with EC, *E. coli *was isolated in 46.2% of patients, whereas *Pseudomonas aeruginosa* was only documented in 7.7% of the patients [17]. This case is unusual due to the involvement of *Pseudomonas* in EC, as this organism is not commonly associated with gas formation in tissues [1]. While septic arthritis typically affects large joints like the knee and hip, the pubic symphysis is a known but rare site following urogenital or pelvic interventions, contributing to the rarity of this case. In diagnosing osteomyelitis of the pubic symphysis and other rare sites, imaging modalities such as MRI and CT are critical.

In addition to this primary case, we discussed two additional patient cases that received a diagnosis of EC. These cases highlighted the variability in presentation and causative organisms associated with this condition. The primary case contributes to existing literature by demonstrating a potential link between carboplatin/paclitaxel chemotherapy and the development of EC and septic osteomyelitis in an immunocompromised cancer patient. Prior studies document carboplatin-induced hematuria with concurrent emphysematous cystitis, and pubic symphysis involvement is extremely rare [12]. However, the improvement of our patient's symptoms following ceftriaxone initiation aligns with existing management strategies for Gram-negative infections, which highlights the importance of early recognition and aggressive antibiotic therapy.

Overall, this case supports current literature that emphasizes early imaging, culture-guided antibiotic therapy, and a high index of suspicion for rare but severe complications in cancer patients undergoing chemotherapy. While the two supporting cases emphasize a pattern of emphysematous cystitis developing in patients undergoing chemotherapy as well, further studies will be needed to better explain the incidence of EC and associated osteomyelitis in patients undergoing carboplatin and paclitaxel treatment regimens.

Some limitations are present within this case report. As a single case report with two supportive cases, the findings are descriptive, making it difficult to establish causality between chemotherapy and the development of osteomyelitis or emphysematous cystitis. Another limitation in this case was due to the limited confirmation of microbiological etiologies, as blood cultures were not performed, and urine cultures were obtained after antibiotics were started. This raises the question of whether *Pseudomonas aeruginosa* was the sole etiology of EC in the patient. The patient’s complex history and comorbidities, such as prior pelvic radiation, obesity, and chemotherapy, present several factors that contribute to the interpretation of the patient’s presentation. Long-term follow-up was also limited by the progression of the patient’s underlying malignancy. Finally, the small number of cases reviewed in this report limits the generalizability of the findings.

## Conclusions

This report highlights the importance of close and frequent clinical follow-up of patients on Carbo-Taxol chemotherapy with atypical side effects such as hematuria. EC is a rare UTI, with mild cases being treated conservatively with antibiotics and bladder drainage. Emphysematous cystitis in combination with osteomyelitis and the involvement of *Pseudomonas aeruginosa* is even more unusual. While rare, *Pseudomonas* involvement should be suspected, especially in patients with other comorbidities such as immunocompromised states and diabetes, with both conditions increasing the risk of developing complicated UTIs. As evidenced by the cases presented in this report, the variability in the presentation of EC ranges from benign to severe. Hence, prompt detection and treatment of atypical but severe complications, such as EC and pubic symphysis osteomyelitis, rely on timely imaging and empirical broad-spectrum antibiotic therapy. Patients with urinary symptoms can also benefit from proper hydration therapy. This report also reinforces the importance of early consultation with surgical teams, especially in cases where any bone involvement is suspected, as early intervention has beneficial effects on patient outcomes. Patients who are not surgical candidates will require an individualized plan to maintain quality of life and reduce suffering. Future research should focus on researching the risk factors, incidence, detection methods, and management of EC in Carbo-Taxol-treated oncology patients.
